# Lightweight visual detection approach for apple ripeness perception in intelligent orchard harvesting scenarios

**DOI:** 10.3389/fpls.2026.1956031

**Published:** 2026-10-05

**Authors:** Mingqing Wu, Jianglei Xiao, Yanjun Chen, Yuxin Xia, Guangjian Ying, Yichuan He, Feng Han

**Affiliations:** 1 Modern Agricultural Engineering Key Laboratory at Universities of Education Department of Xinjiang Uygur Autonomous Region, Tarim University, Alar, Xinjiang, China; 2 Key Laboratory of Tarim Oasis Agriculture (Tarim University), Ministry of Education, Tarim University, Alar, Xinjiang, China; 3 Xinjiang Production and Construction Corps Key Laboratory of Utilization and Equipment of Special Agricultural and Forestry Products in Southern Xinjiang, Tarim University, Alar, Xinjiang, China; 4 College of Biological and Agricultural Engineering, Jilin University, Changchun, China

**Keywords:** apple ripeness detection, complex orchard environment, deep learning, fruit visual recognition, object detection

## Abstract

Apple ripeness detection in complex orchard scenarios is plagued by fruit occlusion, overlapping, intense illumination interference, and subtle maturity feature differences among similar fruits. Conventional lightweight detection models also suffer from low accuracy and severe feature loss in field environments. To address these problems, this paper proposes a real-time lightweight method for apple ripeness detection with optimized multi-dimensional network structures, which balances lightweight deployment and feature perception capability for complex orchard backgrounds. Specifically, depthwise convolutions replace standard convolutions at the backbone tail to reduce redundant computation. A parameter-free SimAM attention mechanism is integrated into the C2PSA module to enhance valid feature extraction of apples under complex backgrounds. Additionally, adaptive downsampling (ADown) is adopted in the neck network to replace traditional downsampling structures, preserving fruit texture and color details and mitigating feature degradation. A multi-category orchard dataset containing red, green, and red-green striped apples is constructed, covering variable illumination, fruit occlusion, and dense stacking conditions. Ablation, comparative, and generalization experiments are conducted to verify model performance. The results show that compared with YOLOv11n, the proposed method achieves 8.6%, 9.5%, 8.9%, 10.9%, and 24.4% improvements in precision, recall, F1-score, mAP50, and mAP50-95, respectively. Its parameters and computational complexity are reduced by 12.8% (2.25 M) and 25.4% (4.7 GFLOPs), with an inference speed of 109 FPS. As a zero-cost feature optimization module, SimAM effectively eliminates classification confusion caused by apples with similar color and appearance. The optimized model presents superior illumination robustness, generalization ability, and convergence performance. It achieves a favorable trade-off between detection accuracy, model size, and inference speed, enabling real-time edge deployment in field orchards. This study provides technical support for intelligent ripeness detection and automatic grading of orchard fruits.

## 1 Introduction

Apples are a popular and economical fruit in the market, with a large-scale fruit tree cultivation industry and a mature and well-established production and distribution system (Amin et al., 2026). Determining fruit ripeness is integral to the entire process—from harvesting and quality sorting to storage, preservation, and market sales (She et al., 2025) (Karmakar et al., 2025) (Olisah et al., 2024) (Chu et al., 2025). The results of ripeness assessment directly impact the timing of harvest, the selection of subsequent processing methods, and the market value of the produce (Wang et al., 2026b). Conducting efficient and precise ripeness testing is of critical practical importance for promoting the standardized and intelligent development of the fruit industry (Li et al., 2025).

Traditional methods of fruit ripeness assessment primarily rely on manual visual inspection, with workers distinguishing fruit maturity levels based on their accumulated experience (Tang et al., 2025a) (Zhao et al., 2024b). This approach has numerous inherent flaws: manual judgment lacks standardized criteria, leading to discrepancies in assessment standards among different operators and resulting in inconsistent outcomes (Feng et al., 2024) (Zhao et al., 2024a). Additionally, manual inspection is inherently slow, making it difficult to quickly screen large volumes of fruit, and labor costs remain high (Rizzo et al., 2023). Natural growing environments are highly variable. Objective factors such as surrounding vegetation, clustered fruit, and fluctuations in sunlight intensity can all interfere with visual observation, further increasing the probability of errors in ripeness identification (Xie et al., 2024) (Xiao et al., 2024). Consequently, this traditional operational model is no longer capable of keeping pace with the development of modern, large-scale fruit production.

Continuous innovations in intelligent technologies have driven steady progress in the field of computer vision (Chen et al., 2025) (Yu et al., 2025) (Wang et al., 2026a) (Liu et al., 2026) (Li et al., 2026) (Wang et al., 2024b) (Wang et al., 2023). Object detection algorithms based on deep learning have gradually found their way into agricultural applications (Kanna et al., 2024) (Dalal and Mittal, 2025) (Song et al., 2025) (Yu et al., 2024) (Khan et al., 2025). Thanks to advantages such as non-destructive testing, high recognition efficiency, and standardized evaluation criteria, they have become the mainstream research direction for fruit condition assessment. By capturing real-time images of fruits using image acquisition devices and then autonomously extracting key features—such as color, skin texture, and morphological contours—through deep learning models, these systems can automatically classify fruit ripeness levels (Huang et al., 2025) (Lin et al., 2024). This effectively addresses the various shortcomings of manual inspection and opens up a new technical pathway for fruit ripeness detection in complex scenarios.

At present, after extensive iterative optimization, object detection algorithms have evolved into two mainstream technical architectures: two-stage detection algorithms and single-stage detection algorithms. The two-stage algorithm features a meticulous overall feature extraction process with clearly defined steps for target localization and classification. It achieves considerable recognition accuracy in conventional detection scenarios and excels at capturing fine-grained features (Gu et al., 2024) (Cui et al., 2024).However, this type of algorithm has a cumbersome network hierarchy and complex internal computational logic (Kamilaris and Prenafeta-Boldú, 2018). It consumes substantial computational resources during operation, and model inference takes a long time, making it difficult to adapt to the requirements of field mobile detection devices and real-time harvesting equipment, resulting in poor flexibility for scene deployment (Xu et al., 2020) (Charisis et al., 2025) (Jiang et al., 2026).

Single-stage detection algorithms eliminate redundant computational steps, adopt an end-to-end processing model, simplify the overall network structure, and significantly reduce model inference time, offering notable advantages in overall operational efficiency (Gupta et al., 2025) (Tang et al., 2025b). Among these, the YOLO series of algorithms, with their simple framework design, excellent real-time detection performance, and strong generalization capabilities, have been widely applied in research on the detection of agricultural and forestry fruits, crops, and field debris, becoming a commonly used foundational model in the field of agricultural visual detection (Kanna et al., 2024) (Liu et al., 2025) (Li et al., 2024).

However, directly applying existing YOLO algorithms to real-world apple ripeness detection scenarios still faces multiple practical challenges. In natural growing environments, the background is cluttered with complex elements; branches and leaves often obscure the fruit, and adjacent fruits are tightly packed and overlapping. This frequently results in the loss of local features, preventing the model from fully capturing valid detection information, which in turn leads to missed or misclassified detections (Liu et al., 2024) (Du et al., 2025). Apples at different stages of ripeness exhibit high similarity in appearance, with a narrow range of color gradients and subtle variations in skin texture. Models struggle to accurately capture these minute differences, significantly limiting the precision of ripeness classification (Yang et al., 2025). Furthermore, to ensure general recognition capabilities, most baseline detection models employ a large number of network parameters, resulting in substantial computational demands and high hardware requirements. This makes it difficult to deploy them on compact, portable smart devices with limited computing power, thereby restricting the practical application of the algorithm in real-world field settings (Sui et al., 2025) (Gao et al., 2025).

To effectively address a series of practical challenges—including interference from complex natural environments, difficulties in identifying subtle features, and limitations in lightweight model deployment—many researchers have continuously worked to improve and optimize basic detection models (Qiu et al., 2025). Most existing optimization approaches focus on enhancing detection performance in a single dimension. Some improved models prioritize recognition accuracy at the expense of computational efficiency, continuously expanding network structures, which increases computational load and slows down real-time detection speeds (Geng et al., 2025); Additionally, some lightweight compression methods excessively prune network modules. While this reduces model size and improves runtime speed, it significantly weakens feature extraction capabilities, ultimately leading to a decline in recognition accuracy. This makes it difficult to balance the relationship between detection accuracy, inference speed, and model size, failing to simultaneously meet the dual demands of detection performance and field deployment.

Balancing practical detection performance with hardware compatibility, this paper designs a lightweight apple ripeness detection algorithm, Apple-YOLO-RD, based on the YOLOv11n model. By integrating the regularities of apple fruit appearance with the imaging characteristics of field environments, the algorithm re-engineers the network architecture, optimizes the feature extraction modules and feature fusion logic, and enhances the model’s ability to detect and identify subtle ripeness features and partially occluded targets. While reducing the model’s parameter size and computational overhead, the algorithm retains its core feature learning capabilities and mitigates detection interference caused by complex environments. The structurally optimized algorithm not only performs image inference quickly to meet real-time detection requirements but also consistently outputs accurate and reliable ripeness assessment results, while remaining compatible with portable devices with limited computational power.

This study constructed a scenario-specific sample dataset, completed algorithm training and performance testing, and validated the comprehensive detection capabilities of the Apple-YOLO-RD algorithm through multiple comparative experiments. The algorithm effectively adapts to complex field planting environments and efficiently and stably performs automated apple ripeness identification tasks. It provides a viable technical reference for similar intelligent applications in the detection of fruit ripeness and the appearance grading of agricultural products, helping the fruit industry steadily advance toward intelligent and efficient operations.

## 2 Materials and methods

### 2.1 Dataset construction

Image acquisition was performed in natural apple orchards of Jiaodong, Shandong Province, China. The experimental variety was Fuji apple. Field images were collected from late August to early October 2024 using an iPhone 14 Pro Max. The photography was conducted at multiple viewing angles and distances to simulate actual orchard monitoring conditions, covering diverse illumination environments and dense fruit scenes. In this study, 769 original orchard images were manually captured on site in 2024. To enhance sample diversity and model generalization capability, an additional 801 valid apple images were selected from public agricultural datasets to form the final experimental dataset. All samples were divided into three maturity levels according to surface color, including fully green apples, red-green mixed apples, and fully red apples. Natural interference such as branch and leaf occlusion, fruit overlap and complex background was retained to fit real field application scenarios. Images with blurry details, overexposure and excessive repetition were eliminated. The remaining qualified images were uniformly standardized in resolution and format for subsequent annotation and model training. The dataset display effect is shown in **Figure 1**.

**Figure 1:**
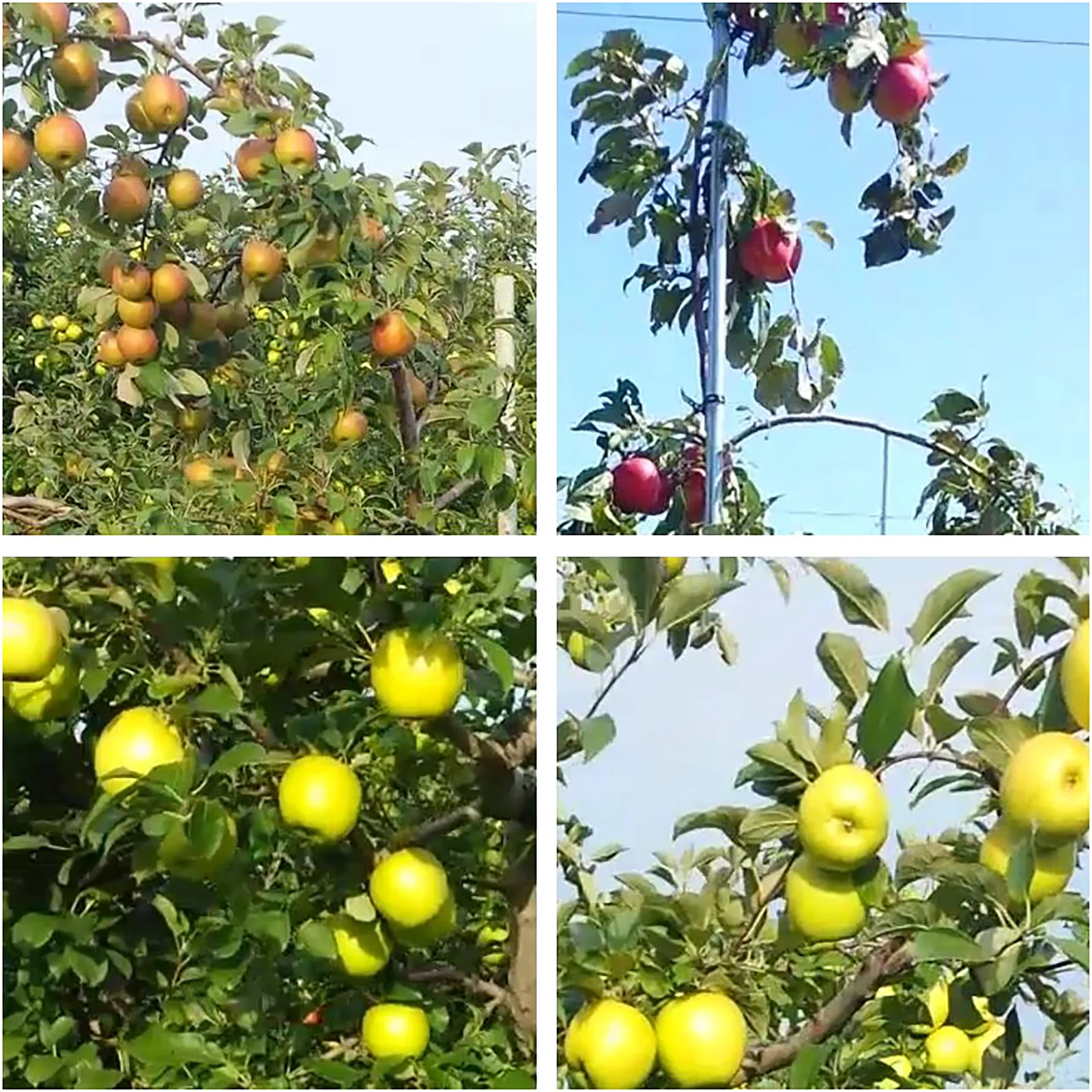
Display of the original dataset.

In this study, the apple maturity classification is solely annotated based on the surface color characteristics of fruits, which is the most intuitive and feasible visual criterion for orchard field detection. However, single color-based labeling cannot completely eliminate ambiguous cases. Specifically, individual differences in fruit pigment distribution, local shadow occlusion, and uneven illumination may cause inconsistent color presentation, which may lead to minor label ambiguity between adjacent maturity categories. In addition, surface color cannot fully reflect the internal sugar content and firmness of apples. Therefore, the current dataset only supports visual appearance-based maturity grading and is not applicable to precise internal quality evaluation. This inherent limitation is retained and further discussed in the limitation section of this paper.

To ensure standardized and repeatable labeling rules for apple maturity grading, this study establishes explicit corresponding relationships between surface color proportion and practical maturity levels. The label division criteria are quantitatively defined as follows. Fully green apples correspond to immature fruits with a green area proportion exceeding 90% of the fruit surface. Red-green mixed apples correspond to mid-mature fruits with a red area proportion ranging from 30% to 90%. Fully red apples correspond to mature fruits with a red area proportion exceeding 90% of the fruit surface. All samples were strictly annotated following the above proportional thresholds to unify labeling specifications.

Target annotation of cleaned images was performed via the professional annotation tool Make Sense following unified criteria. All apple fruits in images were framed based on complete fruit contours regardless of partial branch and leaf occlusion. Three annotation categories were defined corresponding to maturity levels: red apple, green apple and red-green mixed apple. Samples were labeled strictly according to actual surface color to distinguish fruits with different maturity characteristics. Multiple inspections were conducted to revise frame deviation, misclassification and missing annotation, guaranteeing credible labels for model training. Standard annotation files were generated to match images and labels correspondingly. The whole dataset was randomly split into training, validation and test sets at a ratio of 6:2:2. Image sequences were shuffled to evenly distribute three types of samples across subsets and avoid biased partition that impairs model generalization performance.

Diverse data augmentation was applied to training samples to enrich sample diversity and mitigate model overfitting. Weather-related simulation augmentation was adopted to generate images under varied meteorological conditions. Specifically, the brightness adjustment range was set from 0.4 to 1.6 for simulating strong sunlight and cloudy environments. Raindrop overlay with 60–120 random raindrop particles was used to simulate rainy scenes, and random fog noise with opacity ranging from 0.15 to 0.45 was applied to generate foggy scenes. These operations enrich image morphology and light features without altering fruit categories or corresponding annotation labels. The label distribution of the dataset is presented in **Figure 2**. The finalized dataset contains three types of apples with distinguishable features and various field interference and illumination conditions. The annotation results are illustrated in **Figure 3**. Label 0 denotes green apples, label 1 represents red apples, and label 2 corresponds to red-green mixed apples. With complete structure and standardized annotations, the dataset fully reflects the appearance characteristics of apples at different maturity stages. It can sufficiently support the training, validation and testing of the lightweight Apple-YOLO-RD detection algorithm, and meet the experimental data requirements for fruit maturity grading detection.

**Figure 2:**
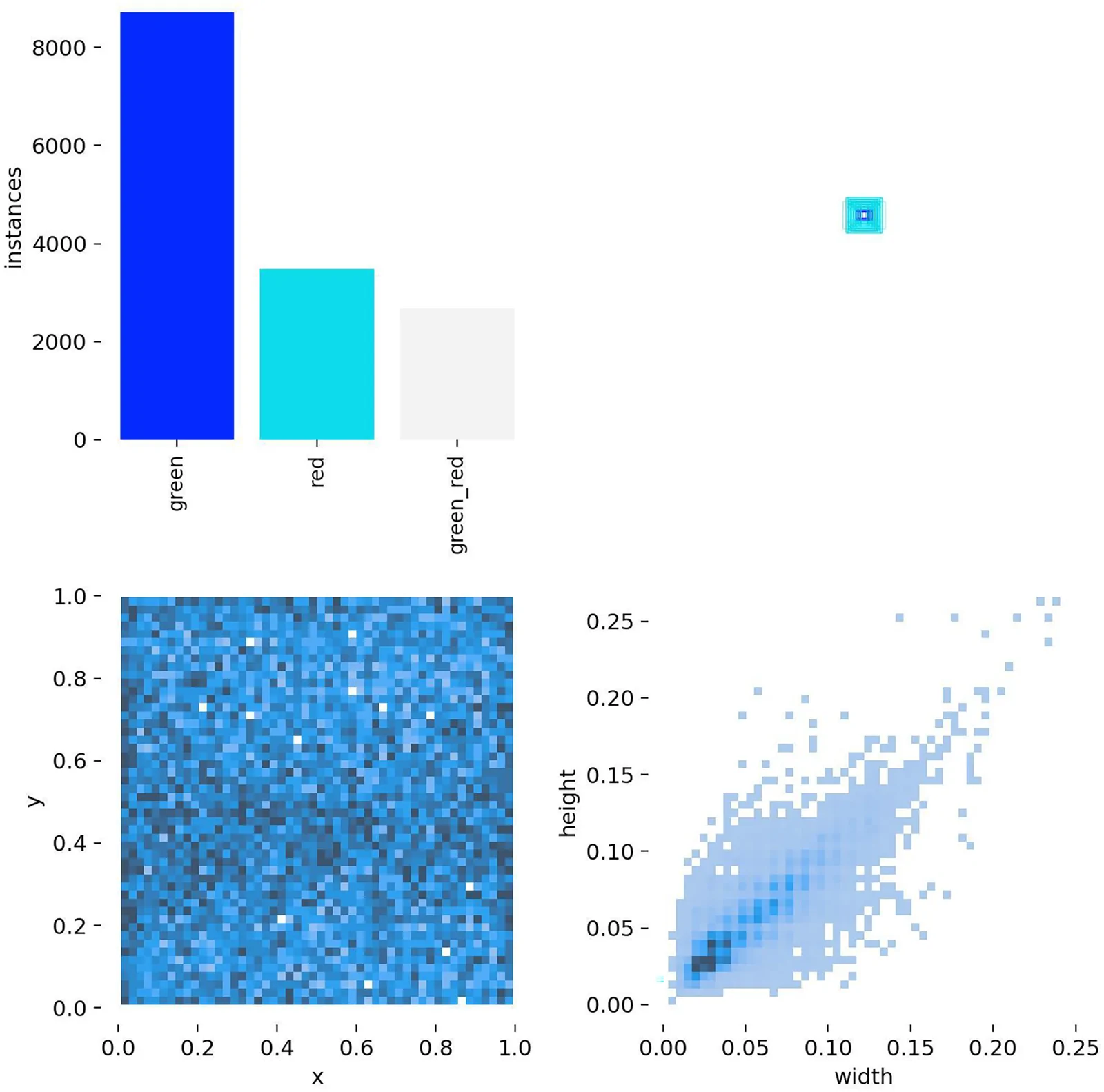
Label distribution of the data set after data augmentation.

**Figure 3:**
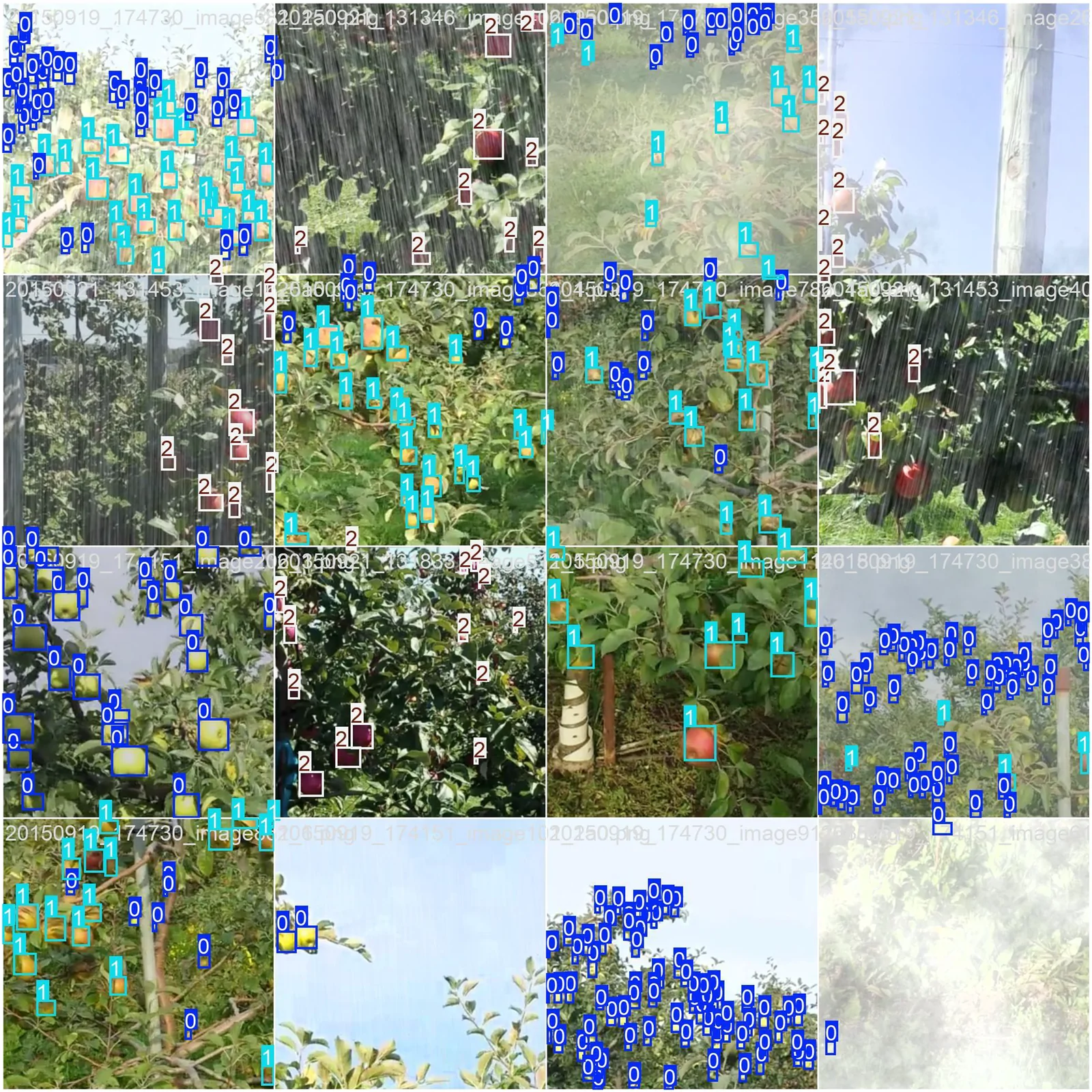
Demonstration of label performance in the data-enhanced dataset.

### 2.2 Model building

#### 2.2.1 YOLOv11n model

YOLOv11n is a lightweight Nano-level detection model released by the Ultralytics team at the end of 2024 (https://github.com/ultralytics/ultralytics). Designed for edge computing devices, it aims to balance detection accuracy and real-time inference speed with minimal parameters and computational cost. It serves as an optimal baseline model for lightweight visual detection in complex farmland scenarios, and fits well in resource-limited applications such as real-time apple maturity detection, mobile terminal deployment and embedded device application. The structural diagram of YOLOv11n is shown in **Figure 4**. It inherits the classic three-stage architecture consisting of backbone, neck and detection head, and achieves lightweight optimization and enhanced feature extraction via newly upgraded core modules with a concise and efficient overall structure. The backbone adopts the novel C3k2 module as the core feature extraction unit. It reduces redundant parameters and computational consumption while retaining multi-scale feature capturing capability, effectively extracting contour, texture and color details to distinguish subtle differences among different apple maturity types. The initial layers implement rapid downsampling through dual convolution to acquire low-level basic features. Equipped with SPPF and C2PSA modules at the tail, the network realizes multi-scale feature aggregation and key region enhancement. It suppresses interference from branches, weeds and cluttered backgrounds, strengthens feature response of occluded and densely distributed fruits, and improves feature extraction stability under complex orchard conditions.

**Figure 4:**
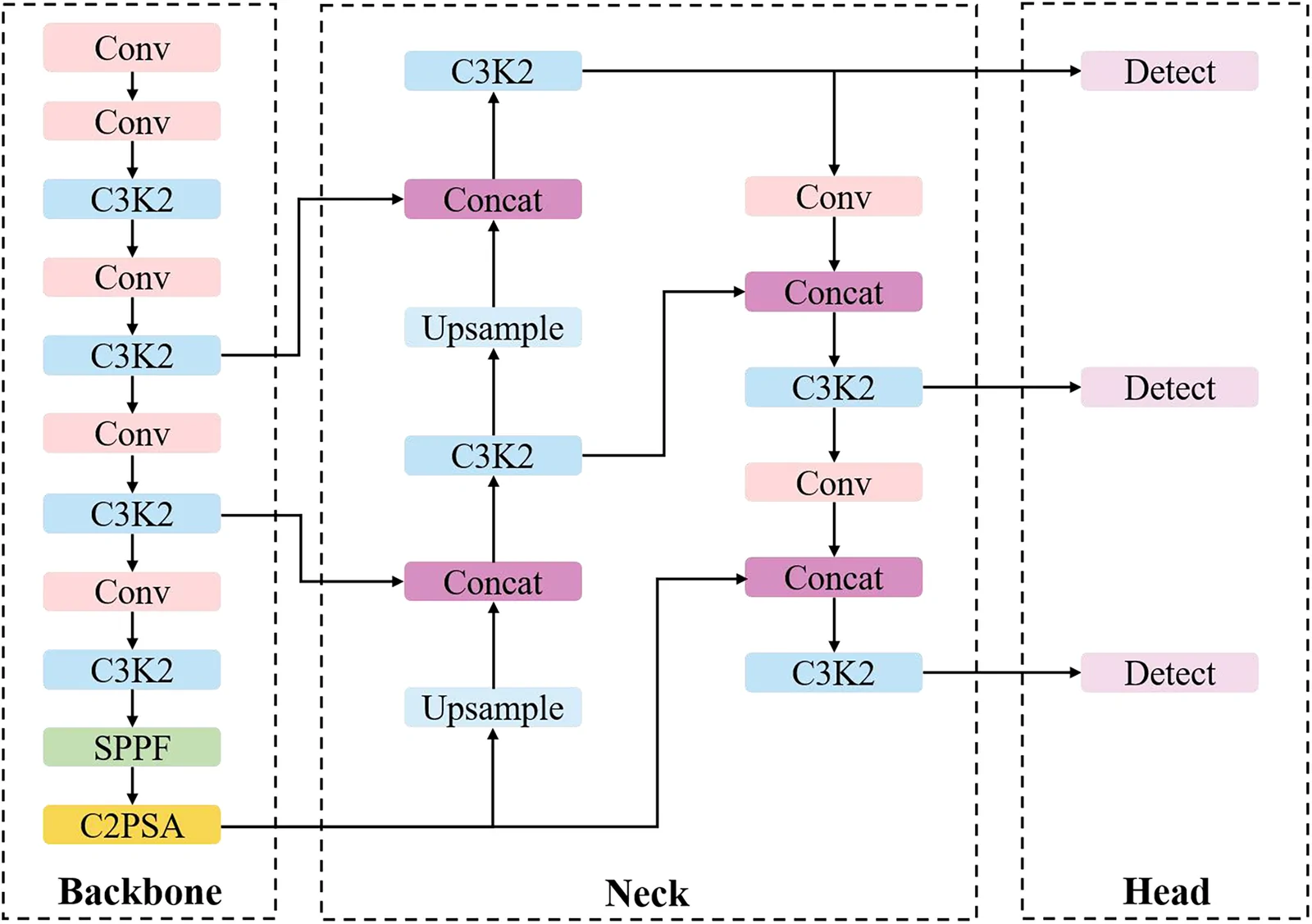
Schematic diagram of the YOLOv11n model architecture.

The neck network adopts the classic FPN+PANet bidirectional feature fusion structure, and conducts multi-dimensional feature transmission and fusion based on lightweight C3k2 modules. The top-down feature pyramid fuses high-level semantic features and low-level detail features to compensate feature loss of small-sized and partially occluded apples. The bottom-up transmission path enhances spatial localization accuracy and alleviates feature confusion caused by dense fruit stacking. This fusion system integrates multi-scale and multi-depth image features without extra computational overhead, retaining distinctive features of apples with different colors and supporting accurate maturity classification.

In the detection head, YOLOv11n employs a decoupled structure optimized by depthwise separable convolution, which separates classification and bounding box regression to eliminate task conflict and feature interference existing in coupled heads. This convolution type greatly cuts down parameters and computation, accelerating inference while maintaining detection precision. Abandoning traditional anchor-based design, it adopts anchor-free detection to adapt to diverse fruit shapes and sizes and avoid matching deviation. Three feature output scales with different strides cover apples of various sizes in complex orchard environments.

For training strategy and performance optimization, the EIoU loss function is utilized to optimize overlap ratio, center offset and aspect ratio error between predicted and ground truth boxes, improving localization accuracy and reducing missed detection and positioning deviation. Compared with previous lightweight models, it achieves optimal trade-off among accuracy, speed and model size, overcoming deployment difficulties of heavy models and weak feature extraction defects of ultra-lightweight models.

Overall, YOLOv11n features lightweight architecture, strong anti-interference capability and superior real-time performance, perfectly matching apple maturity detection tasks. It captures subtle feature differences of apples at diverse maturity stages, copes well with occlusion, overlapping and illumination variation, and suits deployment on low-computing portable devices. As a reliable basic network, it serves as an ideal baseline for intelligent, real-time and precise orchard apple maturity detection, and lays a solid foundation for the improvement of the proposed Apple-YOLO-RD algorithm.

#### 2.2.2 Apple-YOLO-RD model

Based on the lightweight real-time detection model YOLOv11n, this study targets the practical demand of accurate apple maturity detection in complex orchard environments. Aiming at common challenges including branch and leaf occlusion, fruit stacking, light disturbance, subtle color differences among apples of varying maturity and easy loss of small-object features, multi-dimensional optimizations are conducted on the backbone, feature fusion network and feature enhancement module. An improved detection algorithm named Apple-YOLO-RD is constructed with balanced lightweight property, robustness and detection accuracy, and its architecture is illustrated in **Figure 5**.

**Figure 5:**
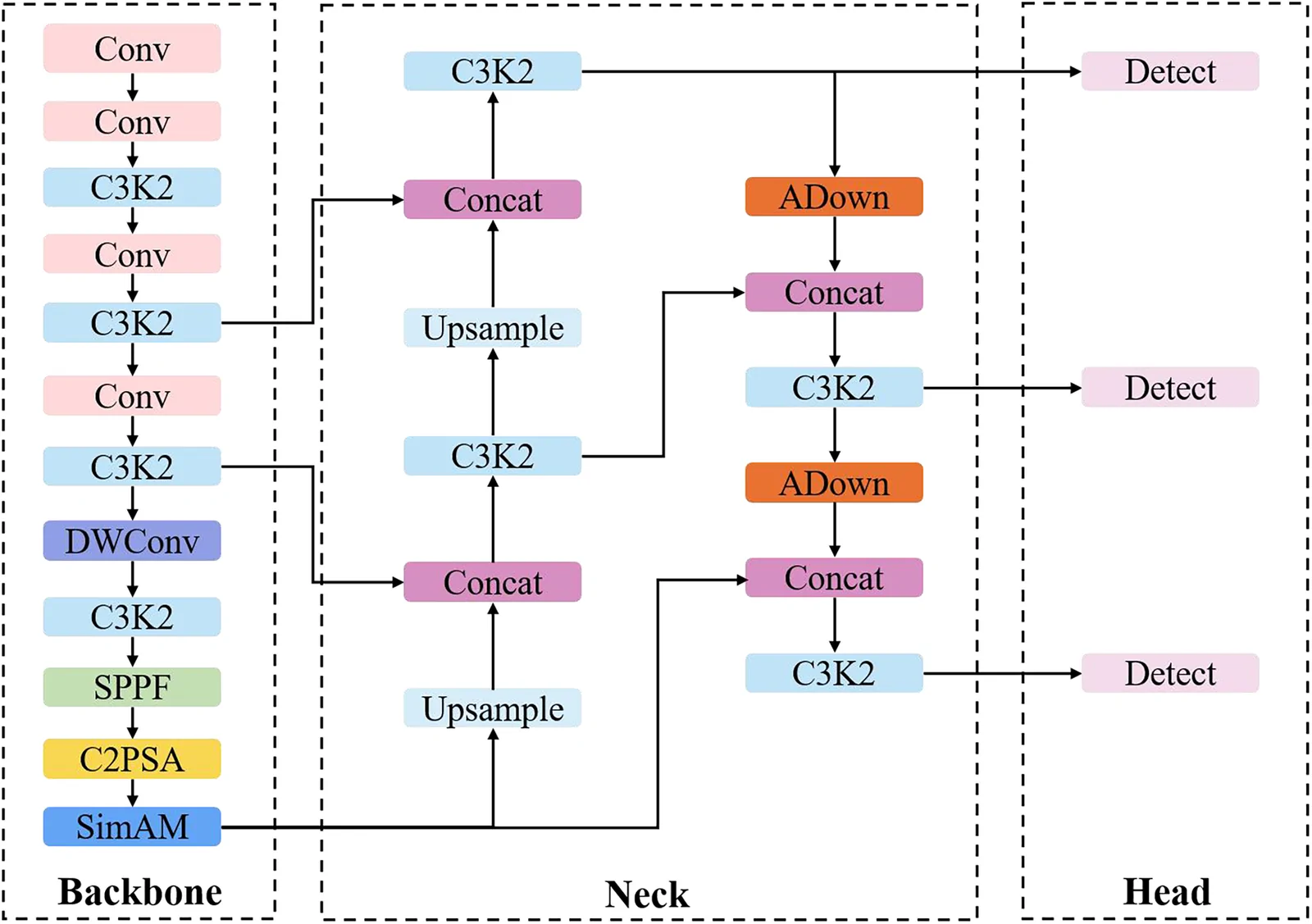
Schematic diagram of Apple-YOLO-RD model structure.

The proposed algorithm maintains the three-stage structure of backbone, neck fusion network and decoupled detection head. It inherits the merits of YOLOv11n such as fast inference speed, low parameter volume and convenient deployment. By means of module replacement and attention mechanism embedding, the inherent drawbacks of the original model including insufficient feature extraction, weak discrimination capability and severe feature loss during downsampling are addressed. The optimized algorithm is better applicable to fine-grained recognition of red, green and red-green mixed apples with different maturity levels.

The backbone network of Apple-YOLO-RD retains the lightweight CSPDarknet architecture and basic C3k2 feature extraction unit. It maintains the lightweight nature and favorable multi-scale feature capturing capability, effectively learning contour, texture and basic color information of apples. To reduce redundant computation and improve deployment adaptability on edge devices, the standard convolution at the last layer of the backbone is replaced with DWConv. This replacement cuts down network parameters and floating-point operations, compresses model size with negligible valid feature loss, and boosts inference efficiency. Let $C_{in}$ and $C_{out}$ denote input and output channels, H and *W* represent feature height and width, and *K* stands for convolution kernel size. The number of parameters in Conv is shown in (Equation 1).

(1) $$K^{2}\times C_{in}\times C_{out}$$

The computational complexity of Conv is shown in (Equation 2).

(2) $$K^{2}\times C_{in}\times H\times W\times C_{out}$$

The number of parameters in DWConv is shown in (Equation 3).

(3) $$K^{2}\times \frac{C_{in}}{G_{in}}\times C_{out}$$

The computational complexity of DWConv is shown in (Equation 4).

(4) $$K^{2}\times \frac{C_{in}}{C_{in}}\times H\times W\times C_{out}$$

It can therefore be concluded that the computational complexity of DWConv is significantly lower than that of Conv; its structural diagrams are shown in **Figures 6C, D**.

**Figure 6:**
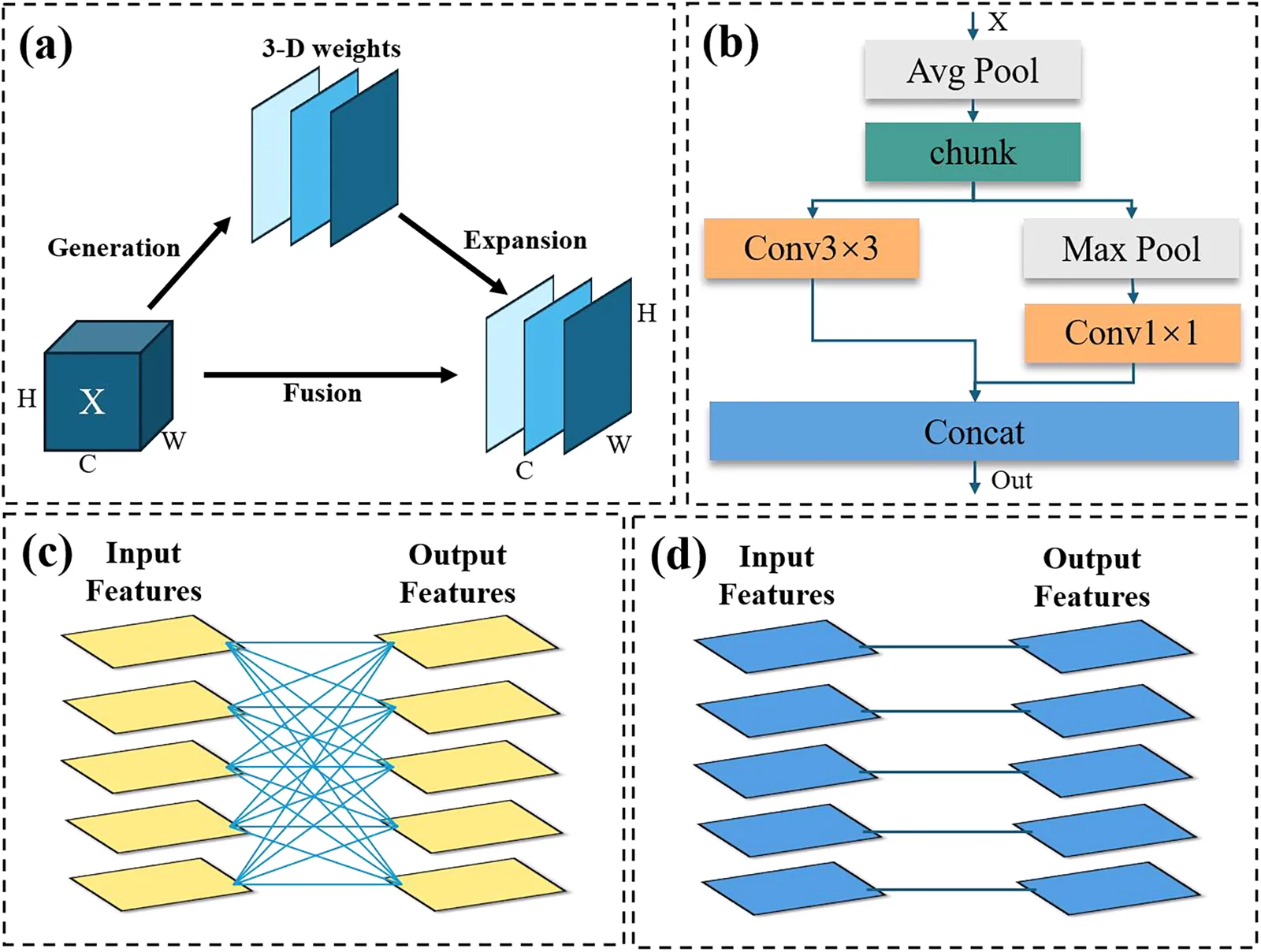
Schematic diagram of modules adopted in Apple-YOLO-RD. **(A)** SimAM attention mechanism, **(B)** ADown, **(C)** Conv, **(D)** DWConv.

Meanwhile, the spatial pyramid pooling module SPPF and spatial enhancement module C2PSA are preserved to aggregate multi-scale fruit features and expand receptive field. To suppress interference from cluttered orchard background and weak target features, the parameter-free SimAM attention mechanism (Yang et al., 2021) is embedded after C2PSA. Inspired by human visual attention, SimAM autonomously generates spatial weight distribution based on internal feature correlation. It adaptively enhances feature response of apple regions and suppresses irrelevant background interference caused by branches, soil and weeds without extra parameters and computation. The module effectively improves perception of occluded, overlapping and red-green mixed fruits, and solves the difficulty in distinguishing subtle maturity features. **Figure 6A** presents the structure of SimAM. As a parameter-free and efficient attention module, it follows the neuroscience principle that informative neurons differ distinctly from surrounding ones. The importance of each neuron is quantified via energy function, enabling adaptive focus on critical areas of feature maps with no additional computational overhead. The core energy function is shown in (Equation 5).

(5) $$e_{t}=\frac{4(\sigma^{2}+\lambda )}{{\left(t-\mu_{t}\right)}^{2}+2\sigma_{t}^{2}+2\lambda }$$

Here, t refers to the feature value of the current neuron. $\mu_{t}$ and $\sigma_{t}^{2}$ represent the mean value and variance of other neurons within the same channel respectively. λ is a tiny regularization constant to avoid zero denominator and ensure numerical stability. A smaller energy value $e_{t}$ indicates a larger difference from surrounding neurons and richer feature information, corresponding to higher attention weight. The energy values are normalized into attention weights via the *Sigmoid* function, as formulated in (Equation 6).

(6) $$A_{t}=\frac{1}{1+\exp(-e_{t})}$$

The final output is obtained by element-wise multiplication of original features and attention weights, which is defined in (Equation 7).

(7) $$\hat{X}=X⊙A$$

Where *A* denotes the final attention weight, *X* represents the original feature map, and ⊙ stands for element-wise multiplication. Requiring no additional learnable parameters, this mechanism can be flexibly embedded into convolutional layers. It enhances key features in both channel and spatial dimensions, meeting the requirements for accurate recognition of subtle differences in apple maturity.

In the neck feature fusion network, conventional downsampling convolution of original YOLOv11n tends to lose shallow detail and color information during scaling, causing feature degradation of small-sized, low-illumination and partially occluded apples, and deteriorating the grading accuracy of apples with different maturity levels. Accordingly, all downsampling modules in the FPN+PANet bidirectional fusion structure are replaced by ADown adaptive downsampling blocks (Wang et al., 2024a). The structure of ADown is shown in **Figure 6B**. Different from fixed-strategy downsampling that damages details, this module adopts adaptive weight learning to compress feature scale while preserving shallow texture, color discrepancy and subtle transitional edge features of three types of apples. Combined with lightweight C3k2 units, the optimized neck network realizes efficient bidirectional transmission and fusion of high-level semantic and low-level detail features. High-level features guide category judgment, while low-level features improve localization precision. The revised structure alleviates feature confusion in dense fruit scenes and achieves complete and stable feature representation for apples of diverse scales and maturity degrees.

The detection head adopts the optimized lightweight decoupled structure of YOLOv11n, which separates classification and bounding box regression tasks to avoid feature conflict and accuracy degradation caused by multi-task learning within a single branch. Equipped with lightweight convolution design, it achieves precise fruit localization and maturity classification with low computational consumption. Combined with the anchor-free detection scheme, the model adaptively matches apples of varying sizes and shapes, greatly enhancing generalization capability in complex orchard environments.

Furthermore, EIoU loss function and diversified data augmentation strategies are applied during training. The positioning error of prediction boxes is effectively optimized. Data transformation covering diverse perspectives, illumination and scales simulates actual complicated field conditions, further improving the recognition stability and anti-interference performance of Apple-YOLO-RD for apples at different maturity stages.

Overall, the proposed Apple-YOLO-RD algorithm achieves simultaneous improvements in lightweight performance, anti-interference ability and subtle feature discrimination via three core optimizations: lightweight convolution replacement at the backbone end, embedding of parameter-free attention module and overall optimization of neck downsampling structure. It retains the baseline model’s merits of fast inference and convenient deployment, and remedies deficiencies including insufficient feature extraction, detail loss and poor anti-interference performance in conventional models. The algorithm can accurately and stably identify and classify red, green and red-green mixed apples, fully satisfying practical demands of on-site real-time detection and intelligent equipment application in orchards.

### 2.3 Evaluation methods

This study evaluates model performance using recall (R), precision (P), F1-score, *AP* and mAP. Taking binary classification as an example: *TP* denotes true positive, *FP* denotes false positive, *FN* denotes false negative, and *TN* denotes true negative. Precision refers to the ratio of correctly predicted positive samples to all predicted positive samples. Recall represents the proportion of accurately identified positive samples among all actual positive samples. mAP is the average value of AP across all categories, and a higher value means better average detection accuracy. The F1-score is the harmonic mean of precision and recall, serving as a comprehensive balanced metric. The corresponding expressions are shown in (Equations 8–12).

(8) $$P=\;\frac{TP}{TP+FP}$$

(9) $$R=\;\frac{TP}{TP+FN}$$

(10) $$F1=\;\frac{2\;\times Recall\;\times Precision}{Recall\;+Precision}$$

(11) AP= ∫P(R)dR ×100%

(12) $$mAP=\;\frac{\sum_{i=1}^{N}AP_{i}}{N}$$

### 2.4 Experimental settings

This study specifies the detailed experimental parameters used for training the proposed network model. The input resolution of images is set to 640×640 pixels, the total number of training epochs is 200, and the batch size is set to 16. AdamW is selected as the optimizer. The initial learning rate is set to 0.01, the weight decay coefficient is configured as 0.0005, and the betas values are 0.9 and 0.999. All experiments are deployed on a computer equipped with an Intel U7-270K Plus CPU and an NVIDIA RTX 5070Ti GPU with 16 GB video memory. The operating system is Windows 11. The software configuration includes Anaconda3-2021.11 Windows version, PyCharm compiler, PyTorch 2.1.2 framework, and Python 3.8.19. All algorithms are implemented and run under identical experimental conditions to guarantee experimental fairness and result comparability.

## 3 Analysis of results

### 3.1 Ablation experiment results

As shown in **Table 1**, a set of ablation experiments are designed to explore the internal working mechanism and synergistic principle of the SimAM attention mechanism, ADown adaptive down-sampling module, and DWConv for apple maturity detection. Targeting the challenges of complex orchard scenarios and subtle feature discrimination of apple maturity, these three modules perform differentiated, complementary and progressive functions and form a closed-loop optimization pipeline. SimAM is dedicated to target feature enhancement. Given that apple maturity discrimination heavily depends on subtle color and texture variations on fruit surfaces, SimAM adaptively suppresses complex background noise and amplifies fine-grained discriminative features of apple objects, which acts as the core component for accuracy promotion. The ADown adaptive down-sampling module focuses on valid feature preservation. It mitigates feature degradation of small-size and overlapping apples induced by conventional down-sampling, so that the delicate maturity features extracted by SimAM can be well retained during deep-layer network propagation. DWConv depth-wise separable convolution is oriented toward lightweight optimization. It removes redundant parameters and unnecessary computational overhead, and frees computational resources for the network to concentrate on learning informative maturity-related features rather than fitting background interferences. Quantitative results of single-module ablation reveal distinct optimization orientations for each component. SimAM delivers prominent accuracy improvement, raising mAP50–95 by 3.7 percentage points and substantially strengthening the model’s capability to focus on subtle apple maturity features. ADown achieves dual gains in accuracy and feature preservation: it increases mAP50–95 by 4.5 percentage points, reduces parameters by 0.14 M and GFLOPs by 1.4, and alleviates deep feature degradation. DWConv mainly pursues model compression; it cuts parameters by 0.18 M with marginal accuracy improvement and effectively reduces computational redundancy. Two-module combination experiments validate remarkable synergistic effects. The overall performance of combined configurations surpasses the simple superposition of individual-module gains. The combination of SimAM and ADown couples feature enhancement and feature preservation, yielding a cumulative mAP50–95 increment of 10.0 percentage points. The combination of ADown and DWConv realizes simultaneous feature retention and network compression, with total parameters reduced by 0.32 M. When the three modules are fully integrated to construct Apple-YOLO-RD, the network establishes a complete positive optimization loop: feature enhancement via SimAM, fine-feature retention via ADown, and efficient lightweight modeling via DWConv. Compared with the baseline YOLOv11n, precision, recall, mAP50 and mAP50–95 are improved by 8.3, 9.5, 10.9 and 24.4 percentage points, respectively. Meanwhile, total parameters decrease by 0.32 M, GFLOPs drop by 1.6, and the inference speed remains nearly unchanged. In summary, the three modules are not naively stacked, but functionally complementary components tailored for apple maturity detection. Their synergistic interaction realizes dual optimization of detection accuracy and model lightweight performance, which fully demonstrates the rationality and superiority of the improvements proposed in this study.

**Table 1:** Comparison of ablation experiment results.

SimAM	ADown	DWConv	P(%)	R(%)	F1(%)	mAP50(%)	mAP50-95(%)	Parameters(M)	Weight (MB)	GFLOPs	FPS (BS = 1)
			86.3	80.9	83.5	84.3	53.8	2.57	5.22	6.3	105
✓			89.5	84.2	86.7	87.6	57.5	2.57	5.22	6.3	104
	✓		88.5	82.7	85.5	86.4	58.3	2.43	4.53	4.9	108
		✓	88.2	83.1	85.6	86.1	57.9	2.39	4.71	4.8	110
✓	✓		91.5	86.2	88.8	89.7	63.8	2.43	4.54	4.9	106
	✓	✓	91.8	87.2	89.4	90.5	68.1	2.25	4.25	4.7	112
✓		✓	91.9	87.4	89.6	90.8	66.1	2.39	4.78	4.8	107
**√**	**√**	**√**	**94.6**	**90.4**	**92.4**	**95.2**	**78.2**	**2.25**	**4.21**	**4.7**	**109**

Bold values represent the optimal experimental result for each evaluation metric within the corresponding table.

### 3.2 Comparison of results before and after model improvement

To further validate the generalization performance of the Apple-YOLO-RD algorithm in complex field environments, this paper compares the apple ripeness recognition performance of the baseline YOLOv11n and the improved model under typical orchard weather conditions, such as sunny, cloudy, and backlit conditions. The specific results are shown in **Figure 7**. The original baseline model demonstrates acceptable recognition stability under well-lit sunny conditions; however, in low-light overcast conditions and backlit scenarios with strong glare, its ability to distinguish the color features of red-and-green-striped apples significantly declines. This leads to frequent misclassifications of ripeness and missed detections of small objects, resulting in poor overall detection robustness. The improved Apple-YOLO-RD, leveraging the feature enhancement capabilities of SimAM attention and the detail preservation advantages of the ADown module, effectively suppresses lighting noise interference under various weather conditions and enhances the ability to extract subtle color and texture features of apples in low-light and high-light environments. Compared to the baseline model, the improved model significantly reduces false positives and false negatives in complex weather scenarios. The recognition accuracy for green apples and apples with mixed red and green hues has improved notably. It can stably adapt to variable outdoor weather conditions, demonstrating stronger environmental adaptability and detection stability, fully meeting the needs for all-weather apple ripeness detection in orchards.

**Figure 7:**
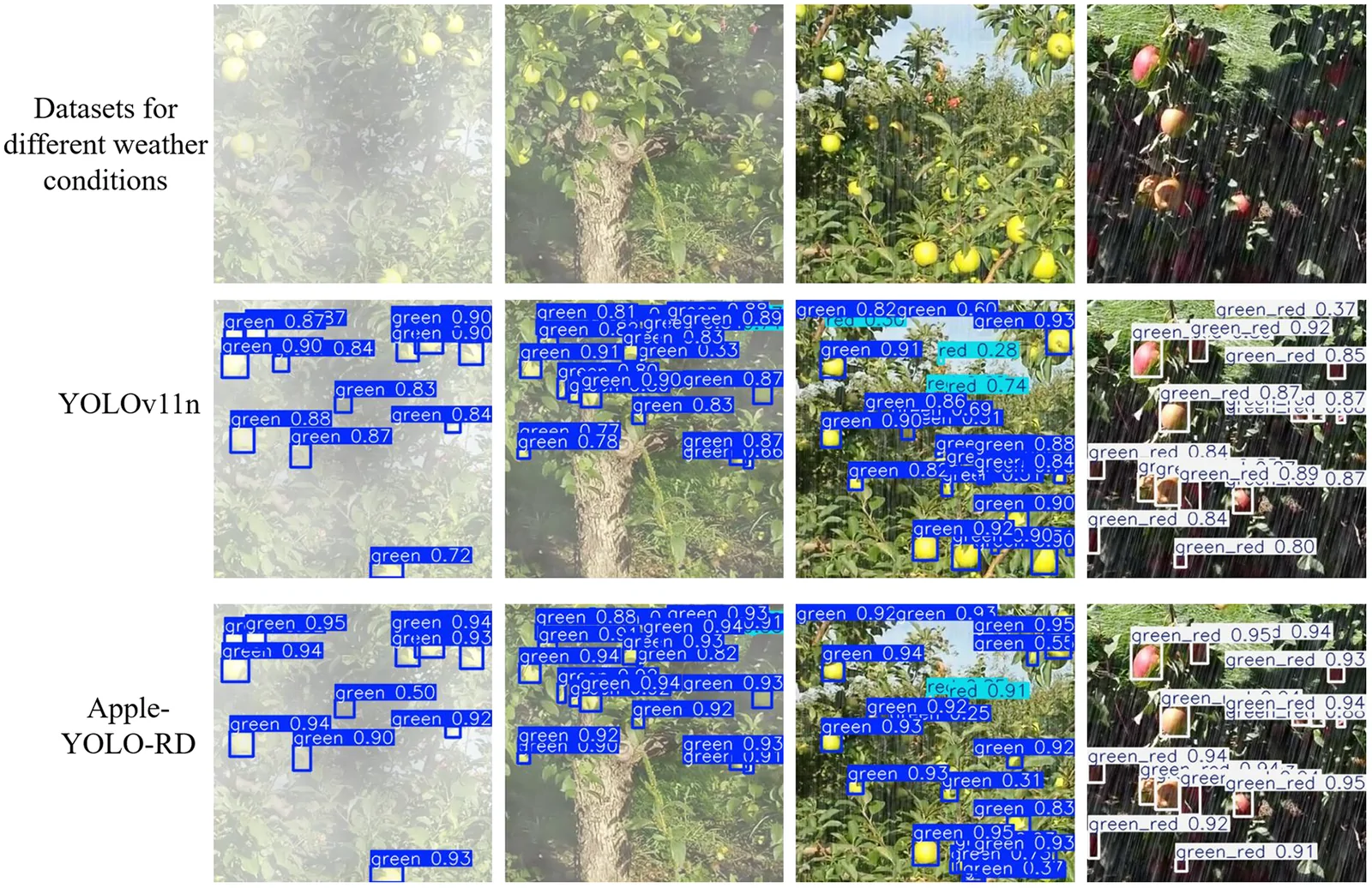
Comparison of recognition performance before and after model improvement under different weather conditions.

**Figure 8** shows performance comparison curves for four metrics—recall vs. confidence, F1 score vs. confidence, precision vs. confidence, and precision vs. recall—before and after model improvement. **Figure 8A** presents the detection results of the baseline YOLOv11n model, while **Figure 8B** shows the detection results of the Apple-YOLO-RD model proposed in this paper. The overall trends in the curves reveal that the baseline model exhibits significant fluctuations across all evaluation curves. At high confidence thresholds, both precision and recall drop markedly, and the curves show poor fit and smoothness. This indicates that the model lacks stability in distinguishing features of red apples, green apples, and red-and-green striped apples, is easily influenced by variations in fruit characteristics, and results in a large number of missed and false-positive detections. The improved Apple-YOLO-RD model exhibits smoother and more robust curves across the board, maintaining higher performance levels throughout the evaluation. It achieves superior precision and recall at various confidence thresholds, with a significant improvement in the F1 score. Additionally, the improved model’s ROC curve exhibits a larger area under the curve and a more stable trend, demonstrating that the model effectively balances detection accuracy and recall performance. It has significantly addressed the baseline model’s shortcomings—namely, weak differentiation of ripeness features and confusion in distinguishing between positive and negative samples—resulting in a comprehensive enhancement of overall detection stability and recognition performance.

**Figure 8:**
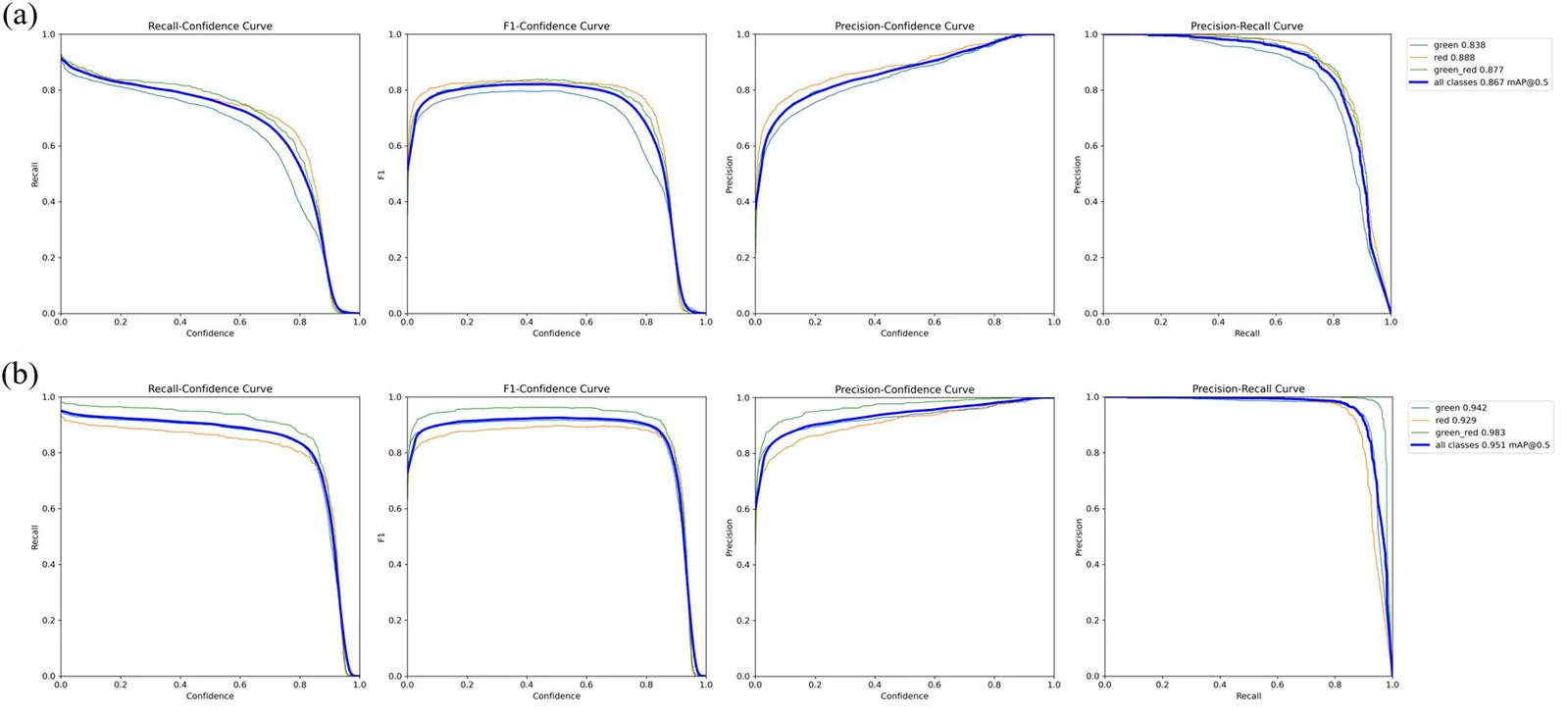
Comparison of recall-confidence curves, F1-Confidence curves, Precision-Confidence curves, and Precision-Recall curves before and after model improvement. **(A)** Before improvement; **(B)** After improvement.

**Figure 9** compares the confusion matrices for multi-class apple ripeness recognition before and after model improvement. **Figure 9A** shows the detection results of the Apple-YOLO-RD model proposed in this paper, while **Figure 9B** shows the detection results of the original YOLOv11n baseline model. The confusion matrices reveal that the baseline model exhibits significant category confusion. Due to the high similarity in appearance between red-and-green striped apples and red or green apples, the baseline model is highly prone to misclassifying these three types of samples. This results in a certain proportion of misclassified maturity levels, accompanied by a small number of missed detections, leading to insufficient overall classification accuracy. In contrast, the improved Apple-YOLO-RD model demonstrates significantly higher recognition accuracy across all categories. It effectively reduces the probability of feature confusion between red-and-green striped apples and solid-colored apples, resulting in more precise classification of red apples, green apples, and red-and-green striped apples. These results clearly demonstrate that the SimAM attention mechanism and the optimized feature extraction architecture can effectively capture subtle feature differences among apples of varying ripeness levels. The ADown module also maximizes the preservation of color transition details, significantly addressing the baseline model’s shortcomings in distinguishing similar features and effectively enhancing the model’s accuracy and reliability in classifying apple ripeness levels.

**Figure 9:**
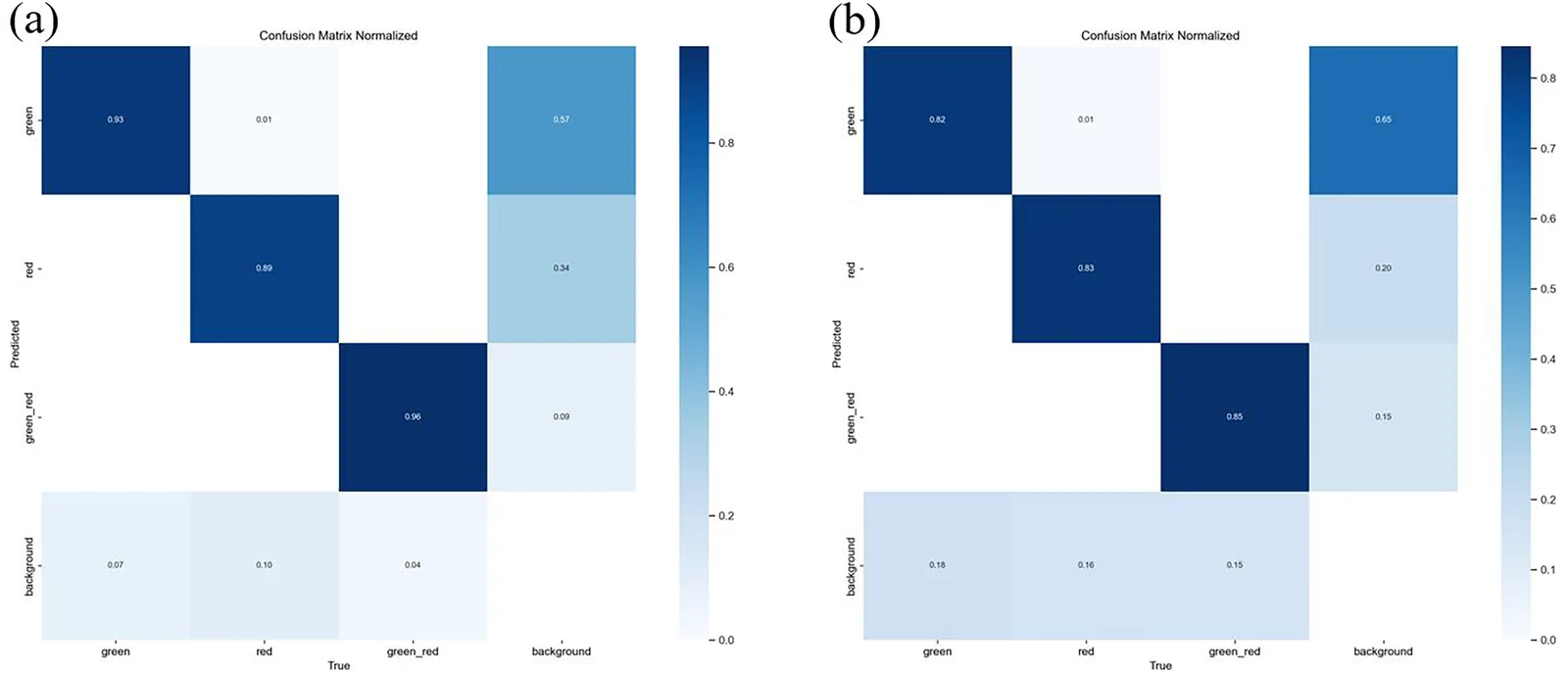
Comparison of confusion matrices before and after model improvement: **(A)** after improvement, **(B)** before improvement.

**Figure 10** compares the training convergence curves before and after model improvement. **Figure 10A** shows the convergence curve of the improved Apple-YOLO-RD model, while **Figure 10B** shows the convergence curve of the original YOLOv11n baseline model. This allows for a visual comparison of the differences in convergence speed and stability between the two models during the training iteration process. The baseline model exhibits a relatively slow rate of loss reduction during the early stages of training. The loss curve shows significant oscillations and fluctuations during the iteration process, indicating an unstable convergence. Furthermore, the final converged loss value is relatively high, suggesting a certain degree of underfitting. This reflects the original model’s low convergence efficiency and poor generalization stability when learning the multi-category ripeness features of apples. In contrast, the improved Apple-YOLO-RD model in this paper exhibits a faster rate of loss reduction during training, achieving rapid convergence in fewer iterations. The training curve remains smooth throughout, with significantly reduced fluctuations, and the final convergence loss value is notably lower than that of the baseline model. The results demonstrate that the DWConv lightweight structural optimization streamlines redundant network parameters and reduces training complexity, while the SimAM attention mechanism and ADown module enhance the quality of feature extraction and fusion. These improvements effectively accelerate the network’s learning of subtle features in red apples, green apples, and red-green striped apples, leading to more stable parameter updates and superior fitting performance. This fully validates that the proposed improvement strategy can effectively enhance the model’s training convergence performance and overall fitting capability.

**Figure 10:**
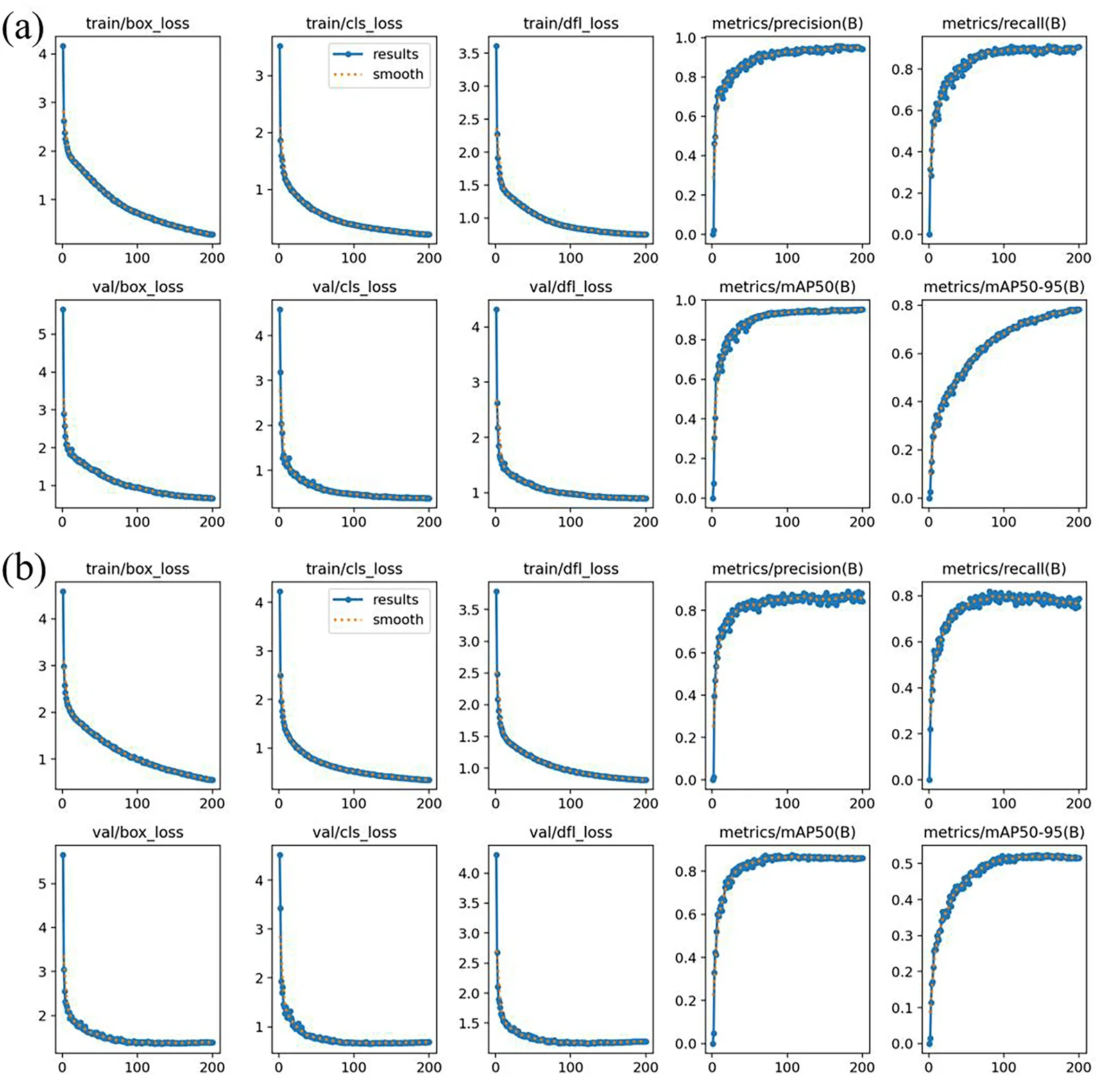
Comparison of training convergence curves before and after model improvement: **(A)** after improvement; **(B)** before improvement.

### 3.3 Comparative experiments with classical models

To fully validate the overall competitiveness of the proposed Apple-YOLO-RD for apple ripeness detection, comprehensive comparative experiments were conducted with multiple mainstream detection algorithms covering different technical paradigms, including two-stage Faster R-CNN-R50, transformer-based RT-DETR-R50, and classical lightweight CNN detectors such as SSD, EfficientDet, and multiple YOLO variants. All models were trained and tested on the same dataset under completely consistent experimental configurations to ensure fair and credible comparison. The quantitative results are summarized in **Table 2**. In terms of detection accuracy and feature discrimination capability, traditional two-stage and early lightweight models exhibit limited performance in complex orchard scenes. The Faster R-CNN-R50 achieves moderate detection accuracy but lacks fine-grained feature perception for subtle apple ripeness differences. The transformer-based RT-DETR-R50 delivers competitive mAP values by virtue of global attention modeling; however, its accuracy gain is still constrained when facing small targets, fruit overlap, and uneven illumination. Classic lightweight detectors, including SSD and YOLOv3-tiny, obtain only 42.8% and 45.1% mAP50-95, respectively, failing to effectively distinguish subtle color and texture differences among apples at different maturity stages. Subsequent YOLO series lightweight models gradually improve overall detection performance, among which YOLOv11n presents the best baseline accuracy with a mAP50–95 of 53.8%. Nevertheless, it still suffers from insufficient feature enhancement and weak anti-interference ability in complex orchard backgrounds. In contrast, the proposed Apple-YOLO-RD achieves a comprehensive and significant performance improvement. Compared with the optimal baseline YOLOv11n, the precision, recall, F1-score, mAP50, and mAP50–95 of Apple-YOLO-RD are increased by 8.3%, 9.5%, 8.9%, 10.9%, and 24.4%, respectively. It outperforms all comparative models across all accuracy metrics, effectively alleviating typical detection difficulties in orchard environments, including ripeness misclassification and insufficient fine feature discrimination. In terms of model lightweightness and real-time performance, Faster R-CNN-R50 and RT-DETR-R50 suffer from extremely high parameter redundancy and computational overhead, resulting in very low inference efficiency. Compared with all lightweight YOLO variants, Apple-YOLO-RD further reduces model scale and computational cost. It only contains 2.25 M parameters with a model weight of 4.21 MB and 4.7 GFLOPs, while achieving a high inference speed of 109 FPS. The results demonstrate that Apple-YOLO-RD effectively balances detection accuracy, model compactness, and real-time capability. Overall, the proposed Apple-YOLO-RD exhibits superior comprehensive performance compared with two-stage, transformer-based, and mainstream lightweight CNN detectors. It is highly suitable for real-time apple ripeness detection in complex orchard environments and supports efficient deployment on edge devices.

**Table 2:** Comparison of different object detection models.

Model	P(%)	R(%)	F1(%)	mAP50(%)	mAP50-95(%)	Parameters(M)	Weight (MB)	GFLOPs	FPS (BS = 1)
SSD	79.3	72.5	75.8	76.1	42.8	14.34	57.36	15.1	64
EfficientDet	83.5	77.2	80.2	80.6	48.5	3.89	15.00	8.4	93
RT-DETR-R50	87.2	81.6	84.3	85.7	55.4	19.83	79.32	31.2	47
Faster-R-CNN-R50	84.8	78.4	81.5	82.5	50.7	41.14	164.56	49.7	19
YOLOv3-tiny	81.2	74.6	77.8	78.3	45.1	12.13	23.20	18.9	72
YOLOv5n	85.2	79.6	82.3	83.1	51.9	2.50	5.04	7.1	96
YOLOv6n	84.0	78.2	81.0	81.5	50.2	4.23	8.29	11.8	86
YOLOv7-tiny	86.1	80.5	83.2	84.0	53.3	4.02	11.70	13.2	73
YOLOv8n	83.7	77.8	80.6	81.2	49.5	3.01	5.96	8.1	98
YOLOv9t	85.8	80.2	82.9	83.6	52.7	1.97	4.45	7.6	85
YOLOv10n	84.6	79.0	81.7	82.2	51.0	2.70	5.49	8.2	84
YOLOv11n	86.3	80.9	83.5	84.3	53.8	2.58	5.22	6.3	105
YOLOv12n	83.3	77.4	80.2	80.8	48.9	2.56	5.28	6.3	92
YOLOv13n	85.5	79.9	82.6	83.4	52.4	2.47	5.19	6.4	101
**Apple-YOLO-RD**	**94.6**	**90.4**	**92.4**	**95.2**	**78.2**	**2.25**	**4.21**	**4.7**	**109**

Bold values represent the optimal experimental result for each evaluation metric within the corresponding table.

To validate the effectiveness and adaptability of the backbone optimization scheme proposed in this paper, we selected several current mainstream lightweight backbone networks for replacement and comparative testing, including classic lightweight networks such as GhostNet, the MobileNet series, ShuffleNetV2, and EfficientNetV2-B0. The test results under a unified dataset and training parameters are shown in **Table 3**. Although various lightweight backbone networks offer certain advantages in parameter reduction, they generally struggle to balance accuracy and speed. Among them, ultra-lightweight networks such as ShuffleNetV2, GhostNet, and MobileNetV3-S have a low number of parameters but limited ability to extract subtle color features of apples, with a maximum mAP50–95 of only 56.1%, resulting in insufficient accuracy for ripeness classification; Networks such as HGNetV2, EfficientNetV2-B0, and VanillaNet show improved accuracy, but their parameter counts and computational demands increase significantly, leading to a substantial drop in inference speed and poor adaptability for edge deployment. Even MobileNetV4-S and StarNet-S050, which offer the best overall performance, achieve mAP50–95 scores of only 62.5% and 69.7%, respectively, failing to meet the requirements for high-precision maturity detection. In this paper, Apple-YOLO-RD leverages an optimized backbone architecture to achieve an mAP50–95 of 78.2% while maintaining a lightweight design with only 2.25 million parameters and 4.7 GFLOPs of computational cost. This represents an 8.5 percentage point improvement over the best-performing baseline backbone networks, while simultaneously achieving an inference frame rate of 109 FPS, thereby achieving a triple optimization of accuracy, lightweight design, and real-time performance. This fully demonstrates that the optimization strategy of our proposed backbone—combining DWConv with SimAM attention—is better suited for multi-class apple ripeness feature extraction tasks in complex orchard scenarios compared to existing general-purpose lightweight backbone networks, offering stronger feature perception capabilities and greater engineering value.

**Table 3:** Comparison of different backbone network models.

Model	P(%)	R(%)	F1(%)	mAP50(%)	mAP50-95(%)	Parameters(M)	Weight (MB)	GFLOPs	FPS (BS = 1)
GhostNet	85.2	81.3	83.2	84.7	56.1	2.13	4.52	4.6	76
HGNetV2	87.5	83.6	85.5	86.9	60.3	3.86	6.44	7.3	87
MobileNetV3-S	84.8	80.7	82.7	83.9	54.8	2.67	5.68	5.8	104
MobileNetV4-S	88.1	84.2	86.1	87.4	62.5	2.32	5.28	2.5	105
ShuffleNetV2	83.9	79.5	81.7	82.6	52.7	2.13	5.40	3.6	112
VanillaNet	86.4	82.1	84.2	85.3	58.6	4.25	4.46	8.6	69
EfficientNetV2-B0	89.3	85.7	87.5	88.8	65.2	5.12	5.48	10.3	76
StarNet-S050	91.7	87.9	89.8	91.4	69.7	2.54	4.23	4.5	95
**Apple-YOLO-RD**	**94.6**	**90.4**	**92.4**	**95.2**	**78.2**	**2.25**	**4.21**	**4.7**	**109**

Bold values represent the optimal experimental result for each evaluation metric within the corresponding table.

### 3.4 A comparative experiment on attention mechanisms

To quantitatively validate the optimal performance of the SimAM attention mechanism in the apple ripeness detection task, this paper embeds mainstream attention modules—including SE, ECA, SAM, CBAM, CA, SK, GAM, and self-attention—into the same baseline network for comparative experiments. The test results under a unified experimental environment are shown in **Table 4**. All types of attention mechanisms can enhance the model’s feature extraction capabilities to some extent and improve the accuracy of apple ripeness recognition. However, most modules suffer from limited accuracy improvements, parameter redundancy, or significant inference speed degradation. Among them, SAM, CBAM, and self-attention modules have relatively high parameter counts and computational overhead, with inference frame rates of only 69 FPS, 71 FPS, and 65 FPS, respectively. Their poor real-time performance makes them difficult to adapt to real-time detection scenarios in the field; Lightweight attention modules such as SE, ECA, and CA feature simple architectures but have limited feature enhancement capabilities, with a maximum mAP50–95 of only 56.1%, and perform poorly in distinguishing subtle features of red-and-green striped apples; the SK and GAM modules show slight improvements in accuracy but still exhibit minor computational redundancy. In contrast, the SimAM attention mechanism demonstrates significant comprehensive advantages. Without introducing additional parameter overhead, the model achieves precision, recall, F1 score, and mAP50–95 of 89.5%, 84.2%, 86.7%, 87.6%, and 57.5%, respectively, outperforming all other attention modules in terms of accuracy. while requiring only 2.57 million parameters and 6.3 GFLOPs of computational power, with an inference frame rate of up to 104 FPS, balancing extreme lightweight design, high detection accuracy, and excellent real-time performance. The experimental results fully demonstrate that SimAM can more efficiently extract key features of apple targets in complex orchard backgrounds, effectively highlight subtle feature differences among apples of varying ripeness levels, and suppress background interference. Compared to other attention mechanisms, it is better suited for apple ripeness grading tasks.

**Table 4:** Comparison of different attention mechanisms.

Model	P(%)	R(%)	F1(%)	mAP50(%)	mAP50-95(%)	Parameters(M)	Weight (MB)	GFLOPs	FPS (BS = 1)
SE	87.7	82.1	84.8	85.6	55.8	2.62	5.48	6.5	82
ECA	87.9	82.3	85.0	85.8	56.0	2.59	5.36	6.4	102
SAM	87.4	81.8	84.5	85.3	55.4	2.65	6.60	6.4	69
CBAM	88.0	82.4	85.1	85.9	56.1	2.71	5.84	6.7	71
CA	87.8	82.2	84.9	85.7	55.9	2.68	5.52	6.5	93
SK	88.2	82.5	85.2	86.4	56.2	2.83	5.32	6.7	85
GAM	88.3	82.2	85.3	86.1	56.3	2.76	5.44	6.6	94
Self-Attention	88.1	82.7	85.6	86.2	56.2	3.12	5.81	6.8	65
**SimAM**	**89.5**	**84.2**	**86.7**	**87.6**	**57.5**	**2.57**	**5.22**	**6.3**	**104**

Bold values represent the optimal experimental result for each evaluation metric within the corresponding table.

**Figure 11** shows the box-constrained Eigen-CAM feature response visualization results of the baseline YOLOv11n and the improved model embedded with the SimAM attention module, to compare and analyze the feature-focusing capability of the network for apple targets. The first row presents the original orchard images; the second row displays the heatmaps of the baseline YOLOv11n; the third row illustrates the visualization outputs of YOLOv11n+SimAM. The yellow rectangles in the figure represent the predicted apple bounding boxes output by the model, and the heatmap color within each box transitions from blue to red, corresponding to feature activation intensity ranging from weak to strong.

**Figure 11:**
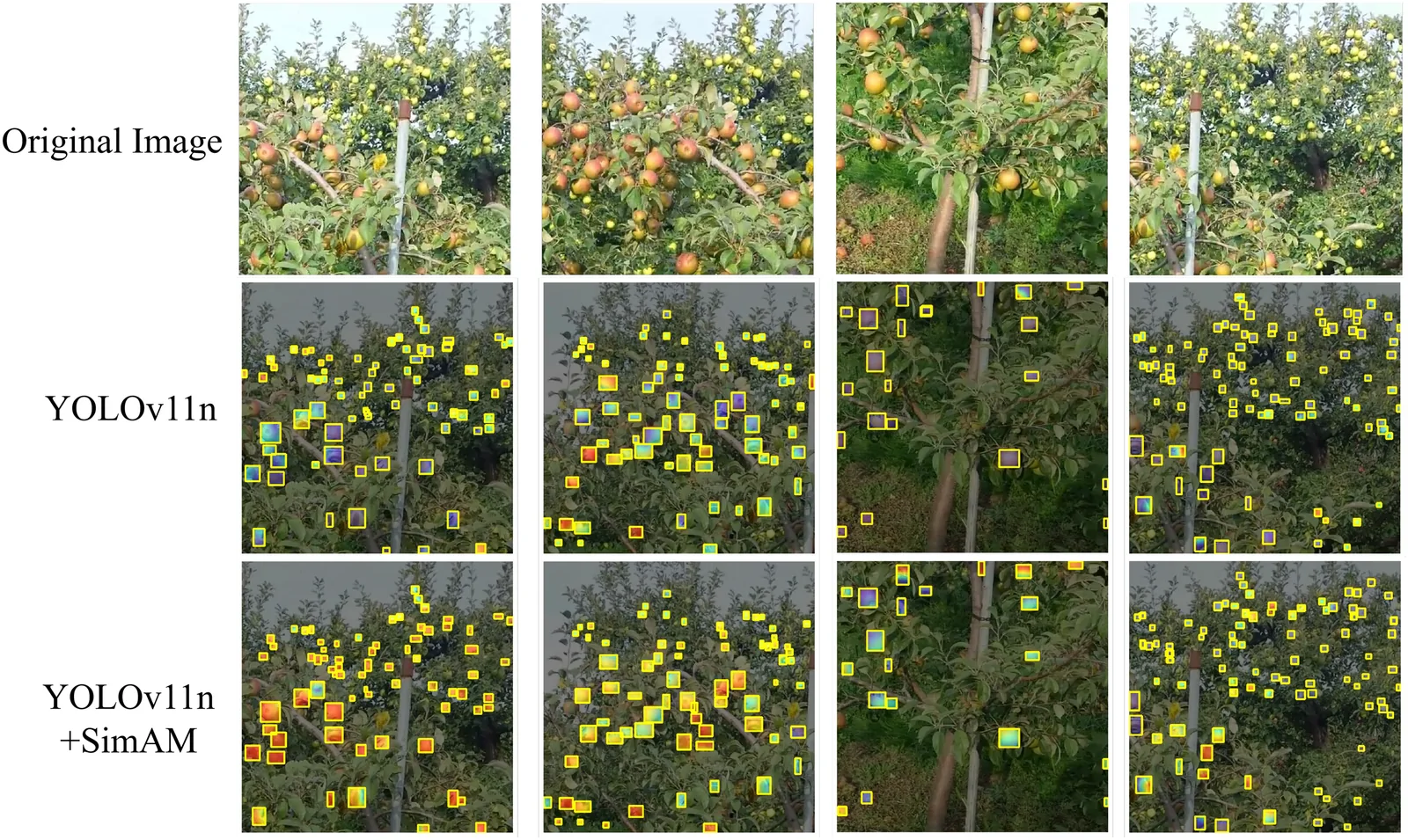
Box-constrained Eigen-CAM visualization of baseline YOLOv11n and YOLOv11n+SimAM. Yellow boxes are predicted apple bounding boxes. Colors from blue to red represent increasing feature activation intensity.

It can be observed from the visualization results that some apple fruits only show weak blue-purple activation responses in the baseline YOLOv11n, indicating insufficient feature responses to target objects. After embedding the SimAM attention module, high-intensity red activations are significantly increased on apple fruit regions. The model can focus more on apple target areas, strengthen the features of fruit bodies, and suppress the interference of invalid background information. It intuitively verifies that the SimAM attention module can effectively improve the model’s feature extraction capability for apple targets.

## 4 Discussion

The Apple-YOLO-RD algorithm proposed in this paper achieves excellent results in the apple ripeness detection task. Through optimizations such as lightweight convolutional replacements, attention embedding, and adaptive downsampling, it effectively enhances the model’s feature extraction capabilities and significantly reduces false positives and false negatives in complex environments. Comparative experiments demonstrate that this algorithm outperforms several mainstream detection models in terms of detection accuracy, model size, and inference speed. It can reliably identify ripe fruits of varying colors and is generally suitable for conventional orchard detection scenarios.

This study still has several limitations. The dataset was collected under limited conditions, with few samples featuring extreme rain or snow, strong backlighting, or extensive fruit occlusion; consequently, the model’s generalization ability in extreme field environments remains insufficient. The study relies solely on external color features to classify ripeness levels, without integrating physical information such as fruit size or flesh quality for a comprehensive assessment, resulting in a relatively simplistic ripeness evaluation system. Additionally, the algorithm is limited to static image detection and has not yet been adapted to dynamic harvesting footage in orchards; its recognition stability decreases when dealing with high-speed moving targets. Furthermore, there is room for improvement in the network architecture’s lightweight design, and computational efficiency in large-scale batch detection scenarios requires further optimization.

To address these shortcomings, future research can be conducted in multiple directions. Continuously expand the sample dataset to include images of special operating conditions, such as adverse weather and dense occlusion, thereby enhancing the model’s resistance to interference. By integrating multi-dimensional evaluation criteria and incorporating fruit morphology and intrinsic quality into grading standards, the scientific rigor of the detection and evaluation process can be enhanced. Concurrently, optimizing the network architecture and inference logic will further reduce computational overhead and improve recognition performance in dynamic scenes. Ultimately, this will facilitate the practical implementation of the algorithm in real-world production processes such as automated harvesting and intelligent sorting, thereby advancing the level of intelligence in orchard fruit inspection. Furthermore, future work could integrate night vision technology to improve the model’s recognition capabilities in low-light conditions.

## 5 Conclusion

This study targets the practical challenges of apple maturity detection in complex orchard scenarios, including frequent occlusion, uneven illumination, low inter-class similarity of immature fruits, and insufficient accuracy of existing lightweight detectors. On the basis of YOLOv11n, this paper proposes an improved lightweight Apple-YOLO-RD algorithm. By introducing DWConv depthwise separable convolution, SimAM attention mechanism, and ADown adaptive downsampling module, the network achieves enhanced feature perception, fine-grained feature retention, and streamlined model structure. Quantitative experiments demonstrate that the proposed method increases the precision, recall, and mAP50–95 by 8.3%, 9.5%, and 24.4%, respectively, while reducing model parameters and computational overhead. The optimized model reaches an inference speed of 109 FPS. Comprehensive comparisons against mainstream CNN-based, two-stage, and transformer-based detection models verify that Apple-YOLO-RD achieves superior overall performance, stable convergence, and strong robustness under variable weather conditions, enabling accurate classification of apple maturity levels. By effectively balancing detection accuracy, model compactness, and real-time capability, the proposed algorithm is well suited for edge deployment in field orchard environments. Future work will focus on further improving the practical applicability of the detection system. First, the dataset will be expanded to cover more extreme weather, dense occlusion, and special growth states to enhance the model’s generalization ability. Second, subsequent research will integrate multi-dimensional information such as fruit texture and internal quality indicators to establish more accurate maturity grading criteria beyond single color features. Ultimately, this study aims to provide technical support for orchard intelligent monitoring and automated fruit sorting systems.

## Data Availability

The original contributions presented in the study are included in the article/supplementary material. Further inquiries can be directed to the corresponding author.
